# Author’s Reply: Rethinking Medical Spanish Competence: Language Proficiency, Clinical Communication, and Inclusion

**DOI:** 10.2196/110709

**Published:** 2026-09-24

**Authors:** Alexandra Lopez Vera

**Affiliations:** 1 California University of Science and Medicine, 1501 Violet St, Colton, CA, 92324, United States, 1 9094980207

**Keywords:** medical Spanish, inclusive communication, LGBTQ+ health, bias, clinical communication, language concordance, standardized patients, simulation training, digital education, curriculum development

I thank the letter writer for this generous engagement with my tutorial [1] and especially for grounding the letter [2] in the lived perspective of a lesbian, gay, bisexual, transgender, queer, plus (LGBTQ+) community member. The letter’s central claim is exactly right: Spanish proficiency and inclusive clinical communication are complementary competencies, not interchangeable ones. A fluent clinician can make a patient feel unseen; an inclusive-minded learner can lack the proficiency to conduct an encounter safely. The tutorial’s formative rubric embeds this distinction by scoring Spanish language effectiveness separately from nonassumptive language, communication repair, and patient-centeredness, so that fluency cannot compensate for exclusion or vice versa [1]. But the letter is correct that formative, module-level tools cannot define readiness for language-concordant care. The field needs standardized anchors.

The proposed three-dimensional model—clinical language proficiency, clinical communication competence, and inclusive communication competence—is a valuable step, and I would frame its payoff in the language of entrustment. The question is not whether a learner speaks Spanish but what that learner can safely be entrusted to do in Spanish and what supports they need. The qualified multilingual assessment [3,4] could anchor the proficiency dimension with verified thresholds, while standardized patient encounters scored with domain-specific rubrics assess the other two. Combined, they could support graduated scopes of practice: a learner might be entrusted to conduct a routine intake in Spanish yet still require interpreter support for goals-of-care conversations or complex counseling, just as procedural skills are entrusted incrementally.

I would extend one of the letter’s questions into a concrete curricular commitment: recognizing when one’s linguistic limits warrant professional interpretation is itself a teachable, assessable clinical safety skill. Programs can operationalize it by adding interpreter-transition phrases to the same phrase banks that teach inclusive language (eg, “Para darle la mejor atención posible, voy a pedir el apoyo de un intérprete profesional”); rehearsing that handoff in simulations, so that it preserves rapport rather than rupturing it; and scoring the judgment itself—not only the language—in assessment rubrics. Framing interpreter use as professionalism rather than deficiency matters doubly for heritage and multilingual learners, who often face implicit pressure to stretch beyond their verified abilities. That is a workforce equity issue as much as a patient safety issue.

Integrating verified proficiency assessment, performance-based inclusive communication assessment, and longitudinal follow-up could move us toward a clinically meaningful definition of language-concordant competence. I thank the letter writer for advancing that agenda and welcome collaboration toward it.

## Abbreviations

LGBTQ+: lesbian, gay, bisexual, transgender, queer, plus
